# One More Step towards a Circular Economy for Thermal Insulation Materials—Development of Composites Highly Filled with Waste Polyurethane (PU) Foam for Potential Use in the Building Industry

**DOI:** 10.3390/ma16020782

**Published:** 2023-01-12

**Authors:** Łukasz Kowalczyk, Jerzy Korol, Błażej Chmielnicki, Aleksandra Laska, Daniel Chuchala, Aleksander Hejna

**Affiliations:** 1 Central Mining Institute, Department of Material Engineering, Pl. Gwarkow 1, 40-166 Katowice, Poland; 2 Łukasiewicz Research Network–Institute of Engineering of Polymer Materials and Dyes, Center for Paints and Plastics, ul. Chorzowska 50A, 44-100 Gliwice, Poland; 3 Faculty of Mechanical Engineering and Ship Technology and EkoTech Center, Gdańsk University of Technology, 80-233 Gdańsk, Poland; 4 Institute of Materials Technology, Poznan University of Technology, Piotrowo 3, 60-965 Poznań, Poland

**Keywords:** recycling, circular economy, composites, polypropylene, polyurethane foam, waste

## Abstract

The rapid development of the building sector has created increased demand for novel materials and technologies, while on the other hand resulting in the generation of a severe amount of waste materials. Among these are polyurethane (PU) foams, which are commonly applied as thermal insulation materials. Their management is a serious industrial problem, due to, for example, their complex chemical composition. Although some chemical and thermochemical methods of PU foam recycling are known, their broader use is limited due to requirements related to the complexity and safety of their installation, thus implicating high costs. Therefore, material recycling poses a promising alternative. The incorporation of waste PU foams as fillers for polymer composites could make it possible to take advantage of their structure and performance. Herein, polypropylene-based composites that were highly filled with waste PU foam and modified using foaming agents were prepared and analyzed. Depending on the foam loading and the foaming agent applied, the apparent density of material was reduced by as much as 68%. The efficient development of a porous structure, confirmed by scanning electron microscopy and high-resolution computed micro-tomography, enabled a 64% decrease in the thermal conductivity coefficient. The foaming of the structure affected the mechanical performance of composites, resulting in a deterioration of their tensile and compressive performance. Therefore, developing samples of the analyzed composites with the desired performance would require identifying the proper balance between mechanical strength and economic, as well as ecological (share of waste material in composite, apparent density of material), considerations.

## 1. Introduction

The era of shrinking global oil and gas resources, which are the primary substrates required for polymer synthesis and as well as a source of the energy necessary to perform this process, has resulted in a significant increase in their price; therefore, it is essential to increase the degree of use of materials that have already been produced. This issue is also critical in the context of limiting the negative impact of humanity on the natural environment. This task can be achieved primarily through material recycling [1,2]. In the case of thermoplastics, currently available recycling techniques, which primarily include material recycling, can enable the technically reasonable and economically viable recovery of the material for reuse. The development of methods for reusing cross-linked plastics remains a significant challenge [3,4,5].

Polyurethane (PU) is one of the most universal polymers, which, depending on the method of obtaining, can be characterized by a wide range of properties, enabling it to be used in many applications [6,7,8,9,10]. Most often (80%), it is used in a foamed form. This form of material is used for thermal insulation and vibration absorption in many industries [11,12,13]. Typical foamed polyurethanes can be categorized as flexible, semirigid, or rigid. The different physical properties of PU foams stem from the type and characteristics of the raw materials, namely polyols and isocyanates [14,15,16]. The management of post-consumer foam waste is a significant technical problem. This type of waste can be deposited in landfills, but this is the worst solution from an economic and ecological point of view. In addition, PU foam waste can be considered safe only in the absence of modifiers in its structure, especially flame retardants (e.g., heavy metals, halides, Sb_2_O_3_), that can migrate to the environment [17,18]. Techniques for the chemical and thermochemical processing of PU foam waste have also been developed and used [19]. Among them should be mentioned alcoholysis, acidolysis, hydrolysis, and glycolysis, which yield raw materials that are possible to process into fully fledged PU materials [20,21,22,23,24]. These methods, however, require significant financial outlays for the construction of safe and effective installations. In addition, a specific limitation to their use is the limited range of reaction products occurring when utilizing PU waste using these methods [25,26,27]. 

Recycling is a worthless technique if it is impossible to use the recovered raw materials. An undoubted limitation of the recycling of foamed plastics, both thermoplastic and cross-linked, is their low density, which makes the transportation of this type of waste to remote disposal installations unprofitable. This is related to administrative and organizational restrictions regarding creating a waste-collecting system. For this reason, many chemical and thermal waste processing installations are located in the vicinity of PU foam production plants, and primarily process technological waste from these units [28,29]. 

Material recycling techniques become more important in the context of the limitations mentioned above. They require relatively small investment outlays, which makes it possible to create smaller, local installations. This reduces problems with the cost and efficiency of waste transport [30,31]. Currently, the material recycling of PU foams primarily involves the removal of impurities and fragmentation. The material is introduced as a filler in new PU foams in this form. In addition, the use of waste reduces the consumption of primary PU [32,33,34]. However, there is a limit to the content of such a filler in the foam, which means that it is impossible to use all of the PU waste that is recovered [35,36,37]. For this reason, searching for new applications for this type of waste is crucial for the recycling of this type of waste.

Expanded polymeric materials are being increasingly widely used. This is primarily due to their favorable mechanical properties, which are able to satisfy the requirements of many applications, and their low weight. Thanks to the significant content of pores in the structure of the product, it is possible to reduce the amount of material necessary for their production, as well as shortening the processing time and energy consumption required for injection molding, because to produce an object, it is only necessary to plasticize a smaller amount of plastic [38,39,40]. The growing popularity of this method of production is also influenced by the development of plastic foaming technology, which makes it possible to obtain objects with a smooth surface and repeatable high mechanical properties resulting from the homogeneous distribution of pores with controlled sizes [41,42]. One way to further improve this class of plastics seems to be the development of foamed plastics that additionally contain fillers that can positively affect the change in the mechanical and functional properties of the material as well as optimize the economic and ecological aspect of processing and use of polymeric materials [43,44,45].

Therefore, the presented research work aims to investigate the application of waste PU foam as filler for polypropylene (PP)-based composites modified with foaming agents. Polypropylene was selected as the matrix for composites due to its exceptional performance, enabling classification as an engineering plastic and a relatively low price [46]. Currently, it is used in the building industry mainly as siding, air and moisture barriers, carpets, and insulating wraps or pipes [47]. Multiple research works have investigated PP application as a component of concrete composites or lightweight aggregates [48,49,50,51,52]. Therefore, selecting PP as a matrix for polymer-based materials for potential application in the building industry is fully justified. The impact of waste foam content, as well as the type of foaming agent, on the structure (apparent density, morphology), mechanical (hardness, tensile and compressive tests), thermal (thermal stability, melting, and crystallization behavior), and insulation (thermal conductivity coefficient) performance of the developed materials was analyzed. 

## 2. Materials and Methods

### 2.1. Materials

**Figure 1:**
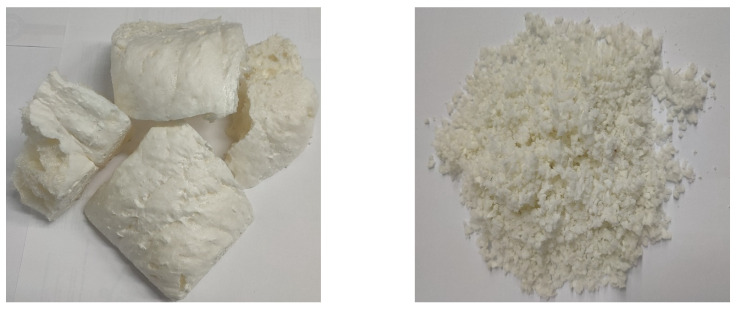
The appearance of PU foam (**left**) before and (**right**) after grinding.

The commercial-grade polypropylene, Moplen HP500N, delivered by LyondellBasell (Netherlands), with a density of 0.90 g/cm^3^ and melt flow rate (MFR) 12 g/10 min (230 °C, 2.16 kg), was applied as a polymeric matrix for the manufacture of the prepared materials. Waste polyurethane foam was applied as filler for the prepared materials. This consisted of wastes generated during the manufacturing of acoustic PU insulation using the spray method, and was characterized by an apparent density of 7–14 kg/m^3^. Before use, the foam was ground using a slow-speed grinder and a sieve with a diameter of 6 mm. Figure 1 presents its appearance before and after grinding. Glycerol obtained from P.P.H. Standard Sp. z o.o. (Lublin, Poland) was used as a compatibilizer for the PP/PU materials. Its purity was 99.5%. Moreover, three blowing agents were applied: GM-0013 (GM) from GM Color Sp. z o.o. (Bydgoszcz, Poland), PLASTRONFOAM C20 (C20) from PLASTRON SAS (Wintzenheim, France) and UNIFOAM MB XPO A50012 (UNI) from Hebron S.A. (Barcelona, Spain). The first two blowing agents were based on sodium bicarbonate and citric acid derivatives, so their decomposition, which yielded the generation of water vapor, carbon monoxide, and carbon dioxide, started at a temperature of around 150–160 °C. The last one, the UNI blowing agent, contained azodicarbonamide dissolved in the polymer matrix, which decomposes at temperatures over 175 °C, generating nitrogen, carbon monoxide, carbon dioxide, and ammonia.

### 2.2. Sample Preparation

To improve the homogeneous distribution of filler and modifiers in PP matrix, all composites were prepared using a twin screw extruder Leistritz ZSE model 27 HP (Nuremberg, Germany). The screw diameter was 27 mm, and the length-to-diameter ratio was 44. The extruder had ten heating and cooling zones. The temperature profile of the extruder was as follows (from the feeding section): 140/145/150/155/160/165/170/175/185/185 °C. The screw rotation during the extrusion processes was 200 rpm, and the throughput was 10 kg/h. 

Due to the low apparent density of the PU waste, its dosing with a gravimetric unit was obstructed. Therefore, materials were prepared using a two-step method. In the first step, PU foam was extruded along with the PP matrix, and glycerol was applied as a compatibilizer to prepare the premix, which was foamed in the second step. The waste foam was dosed into the extruder barrel using the screw side feeders in the 4th zone. Table 1 presents the compositions of premixes prepared in the first step. For comparison, unfilled PP and PP filled with the foam but without the blowing agent were also processed similarly.

During the second step, prepared premixes were extruded with the appropriate amount of blowing agents. All materials were dosed using Brabender gravimetric screw feeders (Duisburg, Germany) with a constant flow rate. Details regarding the compositions are presented in Table 1. For all blowing agents, the reference sample, without PU waste, was prepared with 10 wt% content of modifier, as recommended by the manufacturer. For the application of the UNI blowing agent, the amount was enough to expand extrudate. However, for blowing agents C20 and GM, when waste PU foam was introduced, the amount of modifiers increased to 15 wt%, because the presence of the foam hindered the foaming of the extrudate.

All of the samples were extruded into open, cylindric metal molds, where their volumetric expansion occurred. After they had cooled and solidified, specimens for particular analysis were cut out.

### 2.3. Characterization

The apparent density of prepared materials was determined according to the PN-EN ISO 845:2010 standard as a ratio of the sample weight to the sample volume (g/cm^3^). The samples were measured with a slide caliper with an accuracy of 0.01 mm and weighed using an electronic analytical balance with an accuracy of 0.0001 g. 

The scanning electron microscope (SEM)—HITACHI (Tokyo, Japan) Model SU-3500N, was used in order to assess the structure of the internal surface of samples. The structures of the surfaces of the samples were assessed with an accelerating voltage of 15 kV.

High-resolution computed micro-tomography (µCT) Phoenix v|tome| xs 240 from General Electric Measurement & Control (Boston, MA, USA) was applied to perform 3D observations of the produced samples. The operating parameters were equal to 80 kV for the X-ray source voltage and 360 µA for the tube current. The exposure time was equal to 200 ms. The X-ray projection was recorded with the 360° rotation obtaining 1000 images. The reconstruction (Phoenix Datos 2, General Electric Measurement & Control) was performed with a standard reconstruction algorithm. Each 3D visualization was cut with XY, YZ, and XZ planes in the middle cross-section of each sample to reveal the structure. Based on the reconstructions, three areas with a volume of 3 cm^3^ each were selected from the three-dimensional scanned object. The side surfaces of the analyzed areas were not directly co-located with the cut surfaces of the samples, in order to enable the analysis of volumetric material not subjected to sample cutting processes, which could affect the results of the observations. On the basis of such areas, the porosity and standard deviations of the samples were determined. The quantitative distribution of pore volume, i.e., fractions of pores of different volumes, was analyzed based on obtained histograms.

The prepared materials’ thermal conductivity coefficient (λ) was determined according to the PN-ISO 8301:1998 standard using the HFM 446 heat flow meter from Netzsch (Selb, Germany). Samples with a thickness of 4 cm were tested in the temperature range from 1 to 19 °C using an average temperature of 10 °C. 

The tensile properties were estimated by determining the elastic modulus, tensile strength, and elongation at break. The tests were conducted according to the PN-EN ISO 527 standard, using the Shimadzu tensile testing machine with an elongation head and extensometer. Elongation velocity: 1 mm/min (elastic modulus), 50 mm/min (tensile strength and elongation at break). Number of samples: 5 per test. Trapezium software was used for the evaluation of the test results. 

Shore A hardness test was performed based on the standard PN-EN ISO 868:2005. On the obtained samples, five independent measurements were carried out on the outside wall of the sample and its core. This was aimed at determining the effect of blowing agents on the properties of the material. Since the outer wall of the sample cooled down the fastest, the action of the modifier was the least active there. On the other hand, inside the sample, due to the low thermal conductivity of the foamed polymers, the material had a high temperature for a longer time, and the blowing agent was able to operate for a longer time, creating a more porous structure. Therefore, it was essential to determine the hardness in both of these areas.

Differential scanning calorimetry was performed using DSC1 Star System Mettler Toledo, according to the PN-ISO 11357-1:2016-11 standard. In order to remove the influence of the thermal history on the material, two cycles of heating the sample at a rate of 15 °C/min were performed, after which it was freely cooled to the initial measurement temperature of 25 °C. During the test, the samples were heated to 200 °C, at which temperature the material was kept before cooling for 3 min. To prevent degradation of the polymer, the determination was carried out with a constant flow of nitrogen through the measuring cell at a level of 60 mL/min.

The thermogravimetric (TGA) analysis was performed using the TG 209 F3 apparatus from Netzsch (Selbm, Germany). Samples of foams weighing approximately 10 mg were placed in a ceramic dish. The study was conducted in an inert gas atmosphere—nitrogen—in the range from 30 to 800 °C with a rate of temperature increase of 10 °C/min.

## 3. Results and Discussion

**Figure 2:**
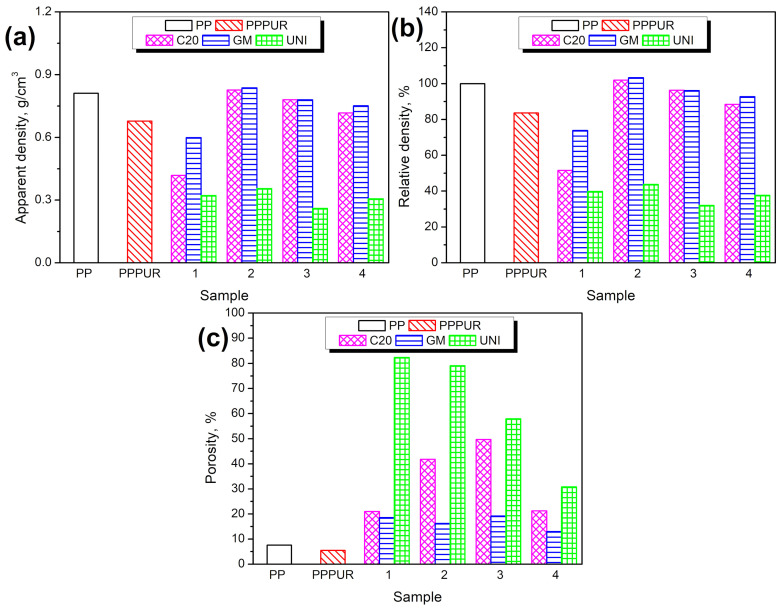
The impact of sample composition on (**a**) apparent density, (**b**) relative density, and (**c**) porosity.

**Figure 3:**
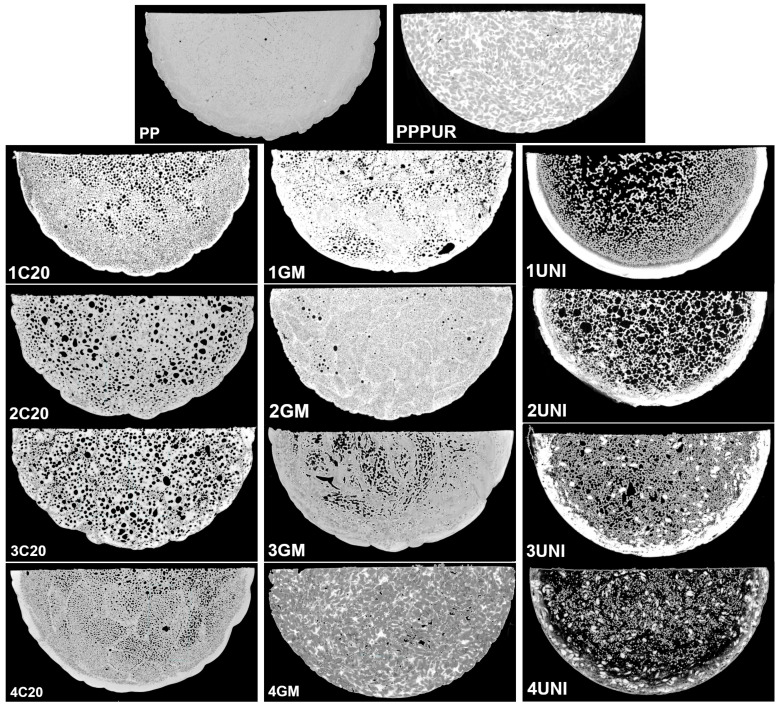
Scans obtained during µCT analysis of prepared samples.

**Figure 4:**
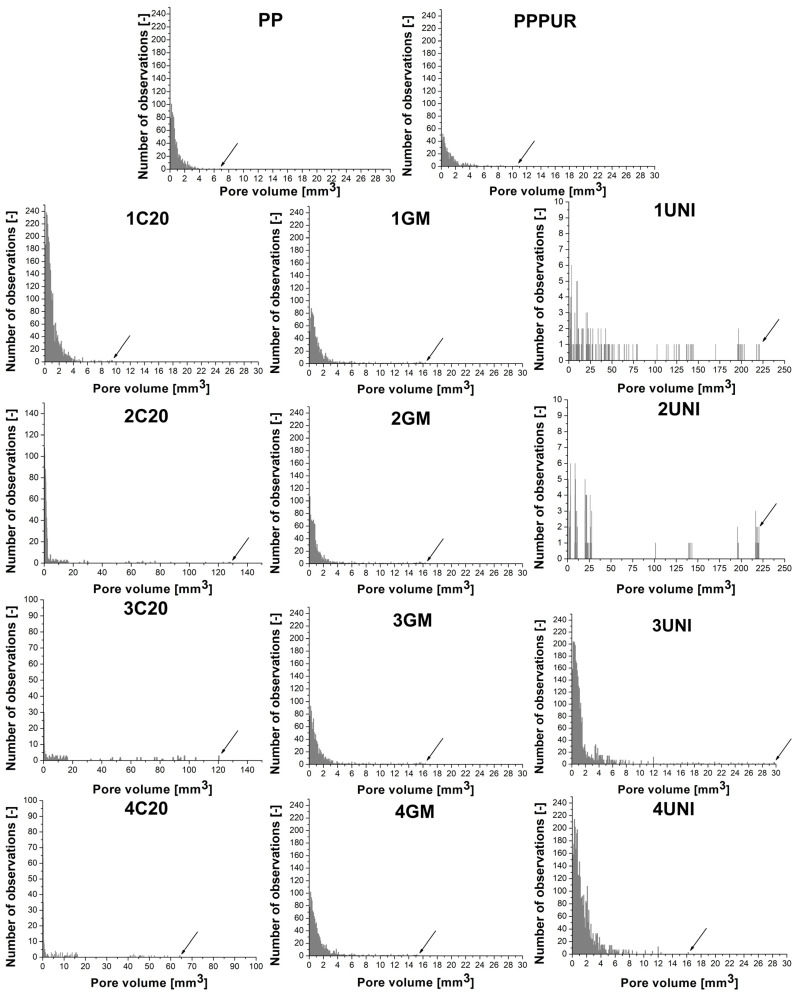
Histograms showing pore volume distribution for prepared samples (arrows indicate the maximum pore volume).

Figure 2 presents the impact of sample composition on the apparent density, relative density compared to unmodified PP material, and porosity of the materials, as determined by µCT analysis. Moreover, images obtained during µCT scans and histograms showing the pore size distribution are presented in Figure 3 and Figure 4. It can be seen that the introduction of waste PUR foam resulted in a decrease in the apparent density of PP, which should be considered beneficial due to the reduced weight and potentially reduced costs during the materials’ lifetime [53,54]. Similar effects were noted for the application of blowing agents. All of them (sample 1) caused a volumetric expansion in PP and a reduction in its density. However, the combination of waste foam with blowing agents was not so effective in all cases. For the lowest PUR content (samples 2C20 and 2GM), a combination with C20 and GM blowing agents led to the relative density of materials exceeding 100%, meaning that the beneficial influence of blowing agents was impeded by the incorporation of foam. This effect can be attributed to the basic character of waste foam, which is related to the chemical structure of urethane ad urea groups [55]. To achieve the most efficient action related to maximum gas generation, sodium bicarbonate requires the presence of acidic compounds [56]. Without them, it only undergoes thermal decomposition, which results in the half production of gaseous compounds [57]. Therefore, the basic character of PU foam may impede the efficiency if C20 and GM foaming agents. Moreover, Sadik et al. [58] reported that the decomposition of chemical blowing agents based on sodium bicarbonate and citric acid, like C20 or GM, is a two-step process with maximum rates at temperatures around 155 and 198 °C. These stages were attributed to sodium bicarbonate and citric acid decomposition, respectively. Therefore, the applied compounds were probably incompletely decomposed, resulting in limited gas generation. On the other hand, the decomposition of the UNI blowing agent begins at a temperature of around 175 °C, which was achieved at later stages of extrusion, so the foaming efficiency was higher. 

With higher loadings of foam, the effect was more beneficial, as a result of the low density of the introduced foam. However, relative density was reduced by only 11.6 and 7.3%, respectively, when using the C20 and GM blowing agents. Significantly better results were noted for the UNI modifier, which caused a decrease in density of over 56% irrespective of PUR content, thus exhibiting the highest efficiency among all analyzed blowing agents. This effect can be attributed to the above-mentioned higher decomposition temperature of the UNI modifier compared to C20 and GM, resulting in the delayed generation of gas, which is removed from the polymer melt in the extruder due to the shear forces acting on the material. The density values suggest that the UNI blowing agent should be considered the most promising among the applied modifiers.

Obviously, the incorporation of cellular PU waste and additional blowing agents affected the materials’ porosity. Scans from µCT revealed significant differences in porosity between the series of analyzed materials related to the different modes of action of the applied additives. Neat unmodified PP showed a homogenous structure with low porosity. The incorporation of waste PU foam induced structural heterogeneity; however, the filler was well dispersed, without noticeable agglomerates, and porosity was not enhanced. For both samples prepared without adding blowing agents, only very small pores were noted, as indicated in Figure 4 by the arrows pointing to the maximum pore volume. The addition of blowing agents significantly changed the number and volume of pores. The highest number of pores was noted for the C20 modifier, which generated a vast number of tiny, well-distributed pores. A relatively similar structure was noted for the GM modifier, but the pore distribution inside the matrix was uneven, pointing to the limited compatibility of the blowing agent with PP [59].

On the other hand, the decomposition of the UNI blowing agent produced a noticeably higher amount of gas, as also suggested by the apparent density measurements. However, the core of the prepared material was characterized by an open-cell structure, which typically has an unfavorable impact on thermal insulation and mechanical performance [60,61,62]. Importantly, for samples prepared without the addition of waste PU foam, the outer layer of materials was solid, without pores, which facilitated trapping the gas inside their structure.

The combined impact of waste PU foam and blowing agent differed between particular modifiers. In the GM compound’s case, the foam incorporation was hardly significant, considering the materials’ porosity. The shape of histograms and the maximum pore volume were almost unchanged. For the C20 blowing agent, the number of observed pores was reduced, while the maximum size of the pores increased due to the coalescence of individual pores, resulting in a partially open-cell structure. This effect can be attributed to the above-mentioned decomposition temperatures of particular components of the blowing agents, resulting in the imperfect utilization of their potential. Moreover, despite their similar active compounds (according to producers, sodium bicarbonate and citric acid derivatives), this effect was not observed with the application of the GM blowing agent. This can be attributed either to differences in the composition of the polymer shells between particular modifiers or to the gas yield. Unfortunately, producers do not provide detailed compositions of materials; therefore, the C20 and GM blowing agents may differ in terms of the applied polymer, or even with respect to its molecular weight, which may affect the melt viscosity and determine the potential for the pores’ coalescence. 

In the case of the UNI compound, the addition of waste PU inhibited the foaming of the composites’ structure. For higher shares of PU foam, the generation of the open-cell structure was limited, probably due to the increased viscosity of the polymer melt, which facilitates the retention of the gas inside the solidifying structure of the polymer [32,63,64]. Sample 3UNI showed noticeably lower porosity compared to the lower PU foam loading, and noticeable closing of the porous structure was noted. This effect was strengthened for sample 4UNI.

The thermal insulation performance of a material is strongly affected by its composition and chemistry, but also by its structure. A porous structure is highly beneficial for insulation performance. Therefore, the most common insulation materials are foams, e.g., polyurethane or expanded polystyrene foams [65]. With respect to polypropylene, it is relatively rarely applied as insulation, but the enhancement of its thermal insulation performance may be beneficial for its multiple applications, for example, in the construction and building industries [66].

**Figure 5:**
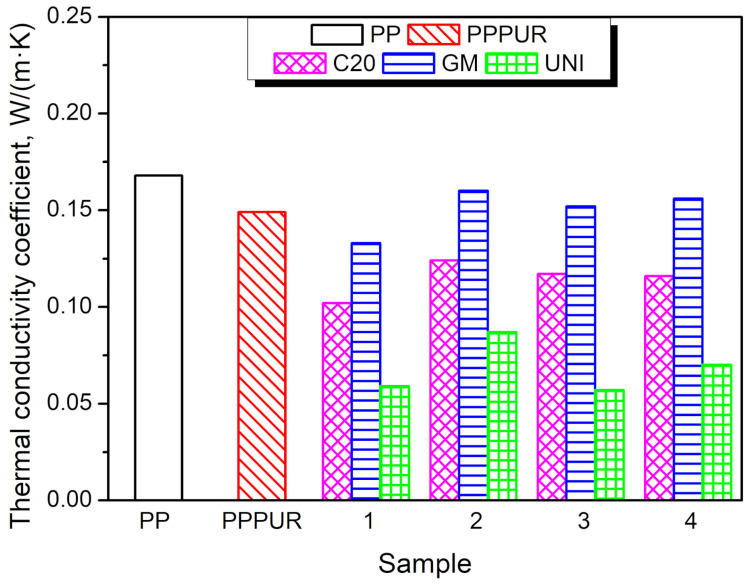
The impact of the applied PP modifications on the thermal conductivity coefficient.

Figure 5 presents the impact of the applied PP modifications on its thermal conductivity coefficient, a parameter that quantitatively describes the insulation performance of the material. It can be seen that the applied modifications of PP reduced the λ value irrespective of the applied formulation. The introduction of the PUR foam caused an 11% decrease in the thermal conductivity coefficient, from 0.168 to 0.149 W/(m·K), which can be attributed to the excellent insulation performance of rigid polyurethane foam. Typically, such materials, commonly applied in the building industry, are characterized by λ values in the range of 0.020–0.035 W/(m·K), which is noticeably lower than those of PP [40]. The incorporation of blowing agents showed a beneficial influence on the thermal insulation performance of prepared materials, which was attributed to the decrease in apparent density caused by the increase in porosity. Increasing the porosity of PP results in the introduction of gases in its structure, either nitrogen, carbon monoxide, carbon dioxide, or ammonia (depending on the blowing agent) generated by the thermal decomposition of blowing agents or air, which may also be present in pores. Among all of the decomposition products, carbon dioxide is present in the highest amounts. The proportion of certain gases is related to whether the pores are open or closed. If the decomposition product of the blowing agents, e.g., carbon dioxide, is not trapped inside the material structure during solidification and escapes, it may yield an open cell structure, thus facilitating the gas exchange with air. Nevertheless, the thermal conductivity coefficients of both gases (CO_2_ and air) are noticeably lower than those of PP, which are 0.015 and 0.025 W/(m·K), respectively, for carbon dioxide and air [67,68]. As mentioned above, sample series 1 (without PU foam) had a solid PP skin without pores, thus enabling the gas to be trapped inside the structure. As a result, these samples exhibited lower thermal conductivity coefficients than composites filled with waste PU foam.

**Figure 6:**
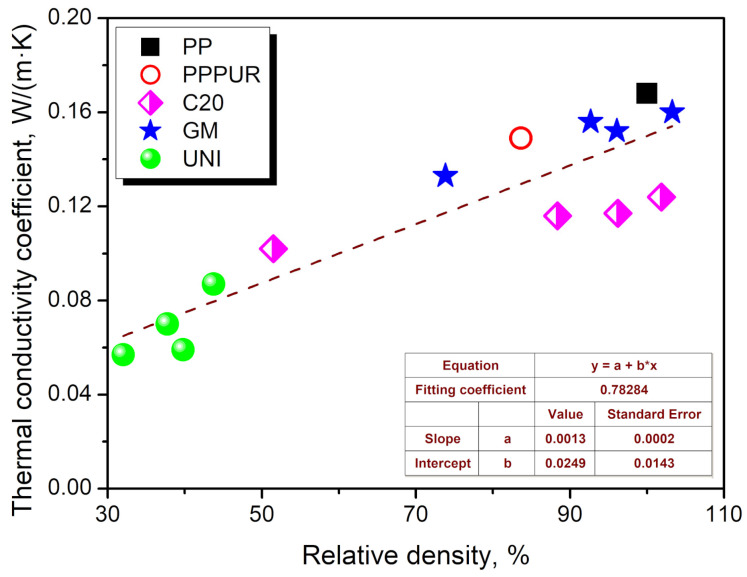
The relationship between λ values and relative density for the prepared samples.

Generally, the apparent density and the resulting porosity play a crucial role in determining the insulation performance of the prepared materials, as can be seen from Figure 6. The presented relationship between λ values and relative density indicates that the decrease in the apparent density of unmodified polypropylene resulting from the incorporation of waste PU foam and blowing agents is highly beneficial for insulation performance. Such relationships are widely known for multiple insulation materials [62].

**Figure 7:**
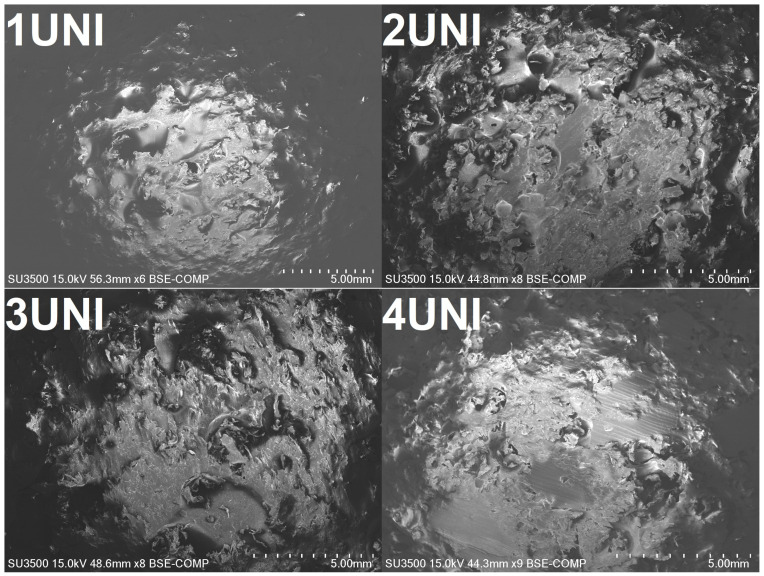
Images obtained from the SEM analysis of the samples modified with UNI blowing agent.

**Figure 8:**
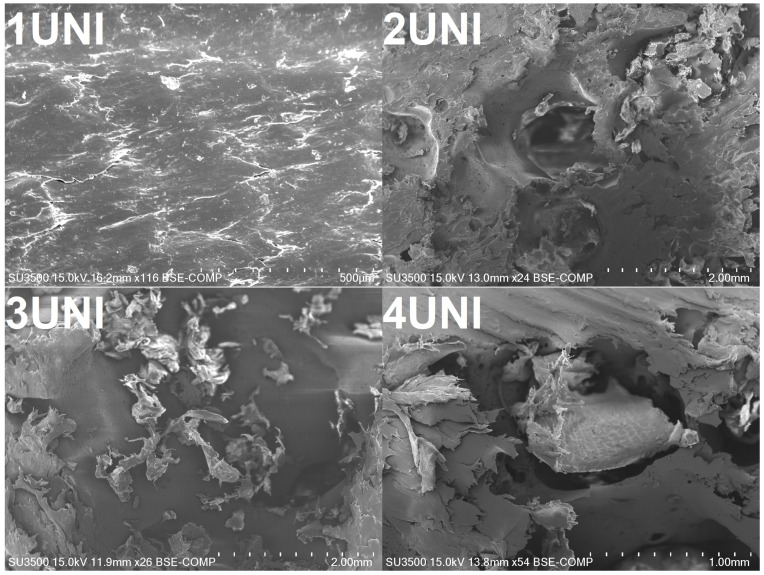
Magnified SEM images of selected samples showing interfacial defects.

Based on the results presented above, the structures of the materials containing the UNI blowing agent, which showed the most beneficial thermal insulation behavior, were investigated using scanning electron microscopy. The obtained images are presented in Figure 7 and Figure 8. It can be seen that all of the samples show highly heterogenous structures with visible pores resulting from the thermal decomposition of the blowing agent, as well as the presence of waste foam. As mentioned above, such a structure can be beneficial to the prepared materials’ insulation performance, resulting in a decrease in the thermal conductivity coefficient.

Figure 8 presents images of the foams taken at higher magnifications, revealing further insights into void formation and the interfacial adhesion between the polypropylene matrix and particles of waste polyurethane foam. The 1UNI sample, which did not contain PU foam, exhibited the most homogenous and smooth surface. On the other hand, the images of the other samples show severe imperfections at the interface, resulting in void generation. This effect is associated with the large difference in polarity between polypropylene matrix and polyurethane foam, as previously reported by other researchers [69,70]. Considering the potential application of the developed materials as thermal insulation, the effect does not have to be indisputably negative. On the other hand, insufficient interfacial adhesion often results in a deterioration in the mechanical performance of polymer composites, which may limit their application range [71].

Table 2 shows the mechanical properties of the obtained materials. Analyzing the results of tensile strength tests, it can be concluded that in the PP samples to which only the foaming additive was added, the strength properties decreased to a lesser extent than in the case of the pure non-expanded polymer. Tensile strength decreased by 28.2–33.9%, depending on the applied modifier. The highest strength was noted for the sample containing the GM blowing agent, while the lowest value was observed for the sample modified with C20. The application of the latter modifier caused the highest weakening of PP performance without the addition of PU foam. On the other hand, UNI, the best blowing agent with respect to density reduction, caused a slightly higher decrease in tensile strength than the GM modifier. This effect can be attributed to its having the highest degree of foaming among the obtained samples, as can be observed in Figure 2 and Figure 3. When analyzing the tensile strength results collectively with respect to the foaming agent used, it can be observed that C20 contributed to the least extent to reducing the strength of the individual samples. On the other hand, for the UNI foaming agent, which was characterized by the best foaming properties among the analyzed additives, the most significant decrease in strength could be observed, which was due to the high porosity of the structure and the reduced proportion of PP in the materials’ volume. 

The incorporation of waste PU foam, into either neat or foamed PP, caused a noticeable deterioration in tensile strength. This effect indicates the limited compatibility of PP and PU as a result of their differences in polarity [72,73,74]. With increasing PUR foam content in the tested materials, regardless of the foaming agent used, a decrease in tensile strength was observed. 

In the case of elongation at break, a drastic decrease, about 75%, in elongation was observed after introducing the foaming agent into the PP. After the introduction of PU foam into the foamed material, there was a further decrease in elongation with increasing PU foam content. The most significant drop was noted for the samples with the highest contents of PU foam. The elongation at break decreased most severely, with the samples with the highest contents of PU foam breaking almost immediately after the application of force. This decrease was comparable for all modifiers and levels of PU foam content in the material. This effect can be attributed to the lack of structural continuity and insufficient interfacial adhesion [75,76,77]. Similar to the tensile strength and elongation at break, changes in the modulus of elasticity were noted. Nevertheless, they were not as significant as that for elongation at break. Therefore, it can be concluded that the foaming of PP does not gradually change its stiffness, as also noted in other works [78].

Hardness values are provided in ShA scale in order to show the differences resulting from the application of the foaming agents and the waste PU foam. For neat PP, when the sample showed high homogeneity, similar values of hardness were noted for both the internal and external parts. The introduction of waste PU slightly decreased the hardness, especially in the core layer, which contained a higher share of PU. The outer layer was composed of PP layer solidified after processing. Similar observations were made for samples modified with blowing agents. As presented in Figure 3, a solid external PP layer was observed on all samples, irrespective of the modifier applied. Therefore, external hardness was always higher than internal hardness. Sample hardness decreased with waste PU foam loading, which was attributed to the increasing porosity. Considering the application of different blowing agents, the most significant drop in hardness was noted for the UNI modifier, which is in line with the reported porosity results.

**Figure 9:**
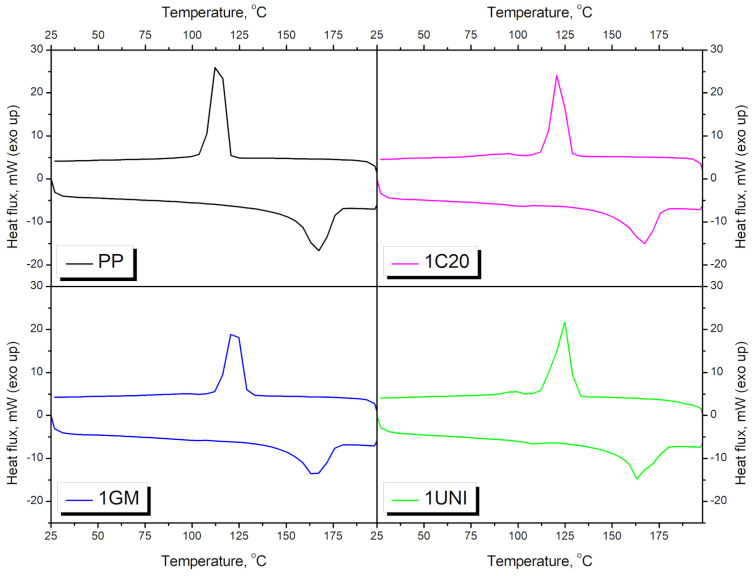
DSC thermograms for neat PP and PP modified with blowing agents.

**Figure 10:**
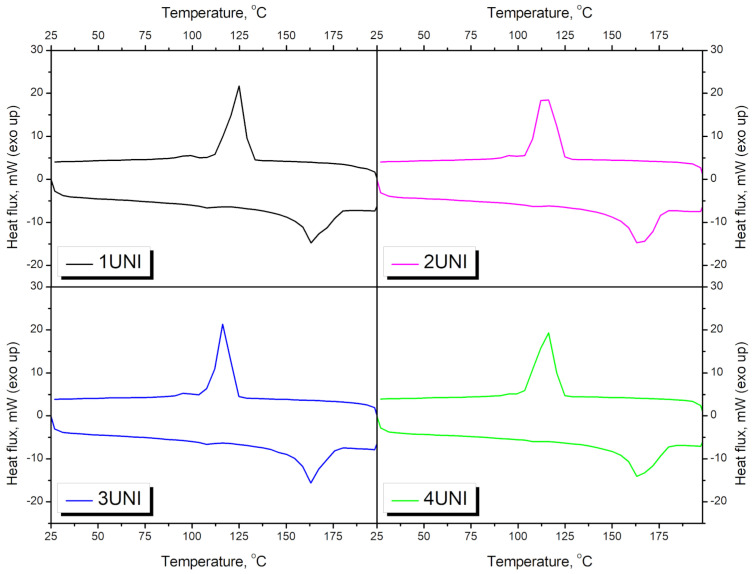
DSC thermograms for neat PP and PP/PU composites modified with the UNI blowing agent.

Table 3 and Figure 9 and Figure 10 present the results obtained from the analysis of the prepared materials using differential scanning calorimetry. The most significant peaks can be observed for polypropylene, the melting and crystallization temperatures of which were determined to be 166.5 °C and 115.5 °C, respectively, which is in line with the literature data [79]. The incorporation of blowing agents caused temperature shifts in the PP peaks, indicating changes in the crystalline structure. A slight decrease in the melting temperature (T_m_) by 0.7–3.5 °C was noted, indicating reduced crystallite size. This effect can be attributed to the impeded PP crystallization induced by the applied modifiers [80]. On the other hand, the application of blowing agents significantly increased the crystallization temperature (T_c_) of PP from its initial value of 115.5 °C to 123.1–126.4 °C, depending on the modifier. This effect indicates that crystallization commences more quickly after the addition of blowing agents, suggesting nucleating activity [81]. Therefore, it can be concluded that the generation of pores inside the material accelerates the crystallization of polypropylene, but the presence of pores significantly affects the size of the generated spherulites [82].

Figure 10 presents the DSC curves of materials foamed with the UNI blowing agent as a function of waste PUR foam content. It can be seen that the main changes can be noted in the case of the T_c_ of polypropylene, which was significantly affected by the addition of blowing agents. Incorporating PUR foam caused a reversion of the effect induced by the blowing agents, indicating the impeded crystallization of the PP macromolecules [81]. This effect was most pronounced in the case of the UNI blowing agent. For lower contents (samples 2 and 3) of the C20 and GM modifiers, the effect was not as significant. The melting of the polypropylene phase was hardly affected by the addition of waste polyurethane foam. Therefore, it can be stated that the incorporation of this waste somewhat affected the rate of crystallization, rather than its degree.

**Figure 11:**
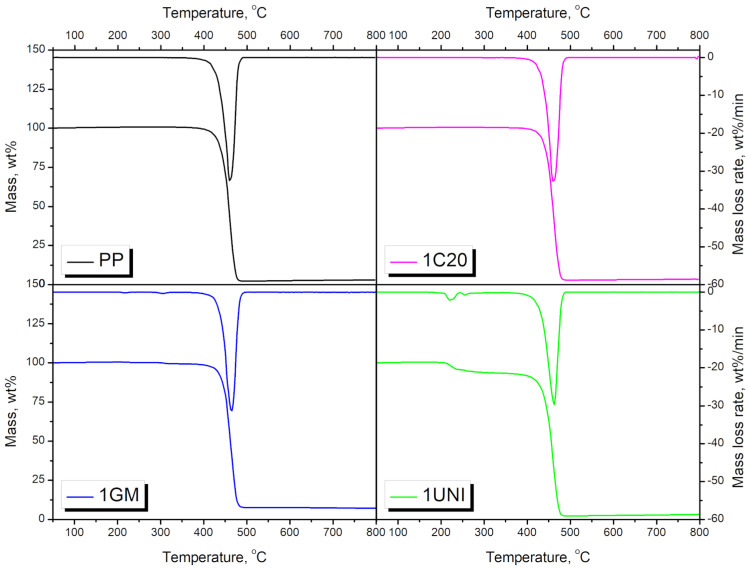
Results of TGA analysis for neat PP and PP modified with blowing agents.

**Figure 12:**
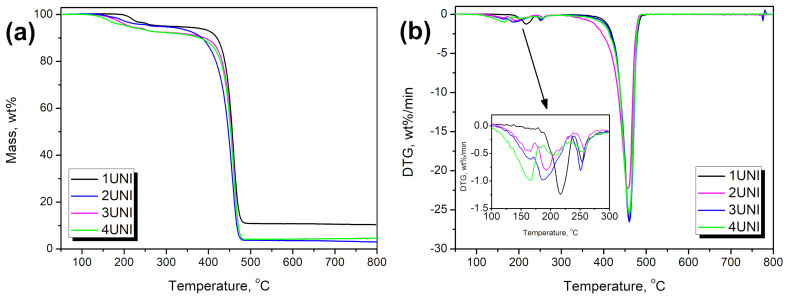
The impact of PUR foam content on the course of thermal degradation of samples modified with the UNI blowing agent. (**a**) mass loss curves, and (**b**) differential thermogravimetric curves.

Thermal stability is an essential feature of materials, often determining their potential window of application. Therefore, Table 4 and Figure 11 and Figure 12 summarize the results of the thermogravimetric analysis performed for the samples. Figure 11 presents the impact of the applied blowing agents on the thermal stability of polypropylene. For unmodified polypropylene, thermal stability, determined as the temperature associated with 2 wt% mass loss, was 413.6 °C. The application of the C20 and GM modifiers resulted in only slight changes in thermal stability, in the range of 412.0–419.5 °C. However, foaming with the UNI modifier significantly reduced thermal stability to 222.9 °C, which can be attributed to with the presence of residual amounts of urea generated during the incomplete decomposition of azodicarbonamide [83].

Figure 12 presents the impact of PUR foam content on the course of thermal degradation of the prepared materials. It can be seen that incorporating this waste results in the deterioration of the thermal stability of composites. This effect can be attributed to the differences in thermal stability of the polypropylene [84] and polyurethane foams [85]. These differences can mainly be observed through the differences in their thermogravimetric curves, which indicate changes in decomposition rate, presented in Figure 12b. These differences are most notable at temperatures between 100 and 300 °C, as a result of the decomposition of urethane groups present in the structure of the PUR foams [64]. Nevertheless, the presented results indicate that the prepared materials are thermally stable at temperatures up to 150 °C, enabling their application at temperatures below the melting point of polypropylene, i.e., 166.5 °C, as determined by the DSC analysis.

## 4. Conclusions

The presented work investigated the possibility of utilizing waste polyurethane foam in the manufacture of polypropylene-based composites, which could potentially be applied as construction materials. Waste PU foam was incorporated to promote PP porosity and reduce its weight and enhance its thermal insulation properties. Nevertheless, the sole introduction of PU foam did not result in a significant performance enhancement. Therefore, composites were additionally modified using blowing agents, whose decomposition during processing yielded gas generation, resulting in the foaming of the materials. The degree of foaming expressed by the decrease in the apparent density and porosity of the prepared materials varied among the applied modifiers, which was attributed to differences in their decomposition mechanisms and temperature. Changes in the materials’ microstructure and morphology were mirrored in the variations of thermal conductivity coefficient and mechanical parameters. Increasing the share of the gas phase in a given volume of material, expressed by a decrease in apparent density, showed a favorable impact on thermal insulation performance, simultaneously resulting in a deterioration in the strength of the materials. Therefore, by adjusting the blowing agent type and loading of the waste PU foam, the balance between insulation and mechanical performance can be shifted, depending on the needs of a given scenario. Importantly, all prepared materials showed thermal stability to temperatures exceeding 150 °C, which should provide a relatively safe window for their application.

## Figures and Tables

**Table 1:** Compositions applied during the first and second step of material manufacturing.

Sample	First Step			Second Step			
	Volume Content, vol%			Mass Content, wt%			
	PP	PUR	Glycerol	Premix	C20	GM	UNI
PP	100.0	0.0	0.0	100	0	0	0
PPPUR	32.5	65.0	2.5	100	0	0	0
1C20	100.0	0.0	0.0	90	10	0	0
2C20	32.5	65.0	2.5	85	15	0	0
3C20	24.2	72.7	3.1	85	15	0	0
4C20	19.3	77.4	3.3	85	15	0	0
1GM	100.0	0.0	0.0	90	0	10	0
2GM	32.5	65.0	2.5	85	0	15	0
3GM	24.2	72.7	3.1	85	0	15	0
4GM	19.3	77.4	3.3	85	0	15	0
1UNI	100.0	0.0	0.0	90	0	0	10
2UNI	32.5	65.0	2.5	90	0	0	10
3UNI	24.2	72.7	3.1	90	0	0	10
4UNI	19.3	77.4	3.3	90	0	0	10

**Table 2:** The results of static mechanical tests performed on the prepared samples.

Sample	Tensile Strength, MPa	Elongation at Break, %	Tensile Modulus, MPa	External Hardness, ShA	Internal Hardness, ShA
PP	31.9 ± 0.2	197.1 ± 29.1	1399 ± 24	98.5 ± 0.7	98.0 ± 0.3
PPPUR	18.8 ± 2.4	6.4 ± 1.3	1263 ± 76	97.8 ± 1.3	96.7 ± 0.2
1C20	21.1 ± 0.9	23.3 ± 5.0	1159 ± 44	98.3 ± 0.9	96.4 ± 0.7
2C20	20.9 ± 0.9	11.4 ± 3.1	1166 ± 35	98.3 ± 0.2	96.3 ± 0.3
3C20	18.4 ± 2.6	5.5 ± 2.3	1053 ± 24	98.0 ± 0.6	94.8 ± 0.1
4C20	13.6 ± 1.6	4.3 ± 2.5	835 ± 11	96.7 ± 0.4	93.2 ± 0.4
1GM	22.9 ± 0.4	20.3 ± 4.2	1365 ± 3	97.6 ± 1.1	97.6 ± 0.8
2GM	16.4 ± 2.0	12.0 ± 1.7	981 ± 24	97.4 ± 0.1	95.5 ± 0.1
3GM	16.4 ± 2.6	7.5 ± 2.3	948 ± 13	95.8 ± 0.2	95.0 ± 0.4
4GM	5.8 ± 0.4	3.6 ± 0.2	722 ± 47	94.4 ± 0.6	92.8 ± 0.2
1UNI	22.2 ± 1.1	21.8 ± 9.7	1162 ± 54	96.0 ± 0.3	81.0 ± 0.3
2UNI	13.5 ± 3.2	2.7 ± 1.3	928 ± 16	96.1 ± 0.9	94.3 ± 1.1
3UNI	9.5 ± 2.1	3.3 ± 1.1	629 ± 60	92.0 ± 1.0	71.0 ± 0.9
4UNI	8.8 ± 2.0	2.6 ± 1.2	527 ± 18	91.8 ± 0.2	81.2 ± 0.7

**Table 3:** Results obtained from the DSC analysis of the prepared materials.

Sample	T_m1_, °C	T_m2_, °C	T_c1_, °C	T_c2_, °C
PP	-	166.5	-	115.5
PPPUR	-	165.8	-	115.8
1C20	101.3	165.8	95.7	123.1
2C20	101.5	163.2	93.7	122.2
3C20	102.5	163.8	92.8	117.9
4C20	102.8	163.7	92.2	118.0
1GM	101.5	164.1	-	124.0
2GM	102.3	164.6	-	123.1
3GM	104.8	166.1	113.6	119.8
4GM	-	168.6	112.3	-
1UNI	99.4	163.0	98.2	126.4
2UNI	99.4	161.8	96.7	118.2
3UNI	99.3	163.8	85.6	115.1
4UNI	100.4	162.8	85.6	116.1

**Table 4:** Results obtained from the thermogravimetric analysis of the prepared materials.

Sample	T_−2%_, °C	T_−5%_, °C	T_−10%_, °C	T_-50%_, °C	Residue, wt%	T_max1_, °C	T_max2_, °C	T_max3_, °C
PP	413.6	425.5	435.0	457.7	3.11	-	-	459.6
PPPUR	187.3	236.4	418.3	461.2	2.98	-	239.4	464.0
1C20	419.5	430.4	438.6	459.2	3.48	-	-	459.8
2C20	200.6	288.5	429.7	459.8	3.42	-	230.3	461.6
3C20	195.3	245.6	429.8	460.9	4.36	-	239.7	462.0
4C20	169.0	234.1	415.4	459.6	3.80	163.0	240.2	463.9
1GM	412.0	430.9	440.7	462.0	7.11	-	-	464.6
2GM	193.1	234.3	423.8	463.9	6.70	-	-	467.7
3GM	162.9	197.5	244.9	463.9	8.48	-	204.1	469.2
4GM	150.1	175.8	215.9	465.2	10.87	173.1	204.4	469.5
1UNI	222.9	258.7	414.1	455.5	3.19	221.3	255.3	462.7
2UNI	190.6	276.1	384.6	447.2	3.01	193.4	256.8	456.1
3UNI	177.9	213.8	391.6	453.9	4.57	187.6	251.1	460.3
4UNI	157.5	208.9	380.0	452.6	4.58	166.8	251.6	459.7

## Data Availability

Data are available in One more step towards a circular economy of thermal insulation materials—development of composites highly filled with waste polyurethane (PU) foam for potential use in the building industry.

## References

[1] Hong M., Chen E.Y.-X. (2019). Future Directions for Sustainable Polymers. Trends Chem..

[2] Thiounn T., Smith R.C. (2020). Advances and Approaches for Chemical Recycling of Plastic Waste. J. Polym. Sci..

[3] Häußler M., Eck M., Rothauer D., Mecking S. (2021). Closed-Loop Recycling of Polyethylene-like Materials. Nature.

[4] Fortman D.J., Brutman J.P., de Hoe G.X., Snyder R.L., Dichtel W.R., Hillmyer M.A. (2018). Approaches to Sustainable and Continually Recyclable Cross-Linked Polymers. ACS Sustain. Chem. Eng..

[5] Liu Y., Yu Z., Wang B., Li P., Zhu J., Ma S. (2022). Closed-Loop Chemical Recycling of Thermosetting Polymers and Their Applications: A Review. Green Chem..

[6] Smoleń J., Olszowska K., Godzierz M. (2021). Composites of Rigid Polyurethane Foam and Shredded Car Window Glass Particles—Structure and Mechanical Properties. Compos. Theory Pract..

[7] Bukowczan A., Hebda E., Michałowski S., Pielichowski K. (2018). Modification of Polyurethane Viscoelastic Foams by Functionalized Polyhedral Oligomeric Silsesquioxanes (POSS). Compos. Theory Pract..

[8] Stachak P., Hebda E., Pielichowski K. (2019). Foaming Extrusion of Thermoplastic Polyurethane Modified by POSS Nanofillers. Compos. Theory Pract..

[9] Małysa T., Nowacki K., Wieczorek J. (2016). Assessment of Sound Absorbing Properties of Polyurethane Sandwich System. Compos. Theory Pract..

[10] Kozioł M., Nowacki K., Wieczorek J., Małysa T. (2015). Evaluation of Mechanical Properties of Polymer Sandwich Systems Used for Noise Reduction Purposes. Compos. Theory Pract..

[11] Augaitis N., Vaitkus S., Członka S., Kairytė A. (2020). Research of Wood Waste as a Potential Filler for Loose-Fill Building Insulation: Appropriate Selection and Incorporation into Polyurethane Biocomposite Foams. Materials.

[12] Abu-Jdayil B., Mourad A.-H., Hittini W., Hassan M., Hameedi S. (2019). Traditional, State-of-the-Art and Renewable Thermal Building Insulation Materials: An Overview. Constr. Build. Mater..

[13] Kurańska M., Barczewski R., Barczewski M., Prociak A., Polaczek K. (2020). Thermal Insulation and Sound Absorption Properties of Open-Cell Polyurethane Foams Modified with Bio-Polyol Based on Used Cooking Oil. Materials.

[14] Stanzione M., Russo V., Oliviero M., Verdolotti L., Sorrentino A., di Serio M., Tesser R., Iannace S., Lavorgna M. (2018). Synthesis and Characterization of Sustainable Polyurethane Foams Based on Polyhydroxyls with Different Terminal Groups. Polymer.

[15] Jiang L., Ren Z., Zhao W., Liu W., Liu H., Zhu C. (2018). Synthesis and Structure/Properties Characterizations of Four Polyurethane Model Hard Segments. R. Soc. Open Sci..

[16] Choe H., Lee J.H., Kim J.H. (2020). Polyurethane Composite Foams Including CaCO3 Fillers for Enhanced Sound Absorption and Compression Properties. Compos Sci. Technol..

[17] Deng Y., Dewil R., Appels L., Ansart R., Baeyens J., Kang Q. (2021). Reviewing the Thermo-Chemical Recycling of Waste Polyurethane Foam. J. Environ. Manage..

[18] Cregut M., Bedas M., Durand M.-J., Thouand G. (2013). New Insights into Polyurethane Biodegradation and Realistic Prospects for the Development of a Sustainable Waste Recycling Process. Biotechnol. Adv..

[19] He M., Gu K., Wang Y., Li Z., Shen Z., Liu S., Wei J. (2021). Development of High-Performance Thermoplastic Composites Based on Polyurethane and Ground Tire Rubber by in-Situ Synthesis. Resour. Conserv. Recycl..

[20] Zia K.M., Bhatti H.N., Ahmad Bhatti I. (2007). Methods for Polyurethane and Polyurethane Composites, Recycling and Recovery: A Review. React. Funct. Polym..

[21] Datta J. (2012). Effect of glycols used as glycolysis agents on chemical structure and thermal stability of the produced glycolysates. J. Therm. Anal. Calorim..

[22] Gama N., Godinho B., Marques G., Silva R., Barros-Timmons A., Ferreira A. (2020). Recycling of polyurethane scraps via acidolysis. Chem. Eng. J..

[23] Kemona A., Piotrowska M. (2020). Polyurethane Recycling and Disposal: Methods and Prospects. Polymers.

[24] Zhu P., Cao Z.B., Chen Y., Zhang X.J., Qian G.R., Chu Y.L., Zhou M. (2014). Glycolysis Recycling of Rigid Waste Polyurethane Foam from Refrigerators. Environ. Technol..

[25] Stančin H., Růžičková J., Mikulčić H., Raclavská H., Kucbel M., Wang X., Duić N. (2019). Experimental Analysis of Waste Polyurethane from Household Appliances and Its Utilization Possibilities. J. Environ. Manage.

[26] Kanchanapiya P., Intaranon N., Tantisattayakul T. (2021). Assessment of the Economic Recycling Potential of a Glycolysis Treatment of Rigid Polyurethane Foam Waste: A Case Study from Thailand. J. Environ. Manage.

[27] Godinho B., Gama N., Barros-Timmons A., Ferreira A. (2021). Recycling of Different Types of Polyurethane Foam Wastes via Acidolysis to Produce Polyurethane Coatings. Sustain. Mater. Technol..

[28] Kiss G., Rusu G., Peter F., Tănase I., Bandur G. (2020). Recovery of Flexible Polyurethane Foam Waste for Efficient Reuse in Industrial Formulations. Polymers.

[29] Shin S., Kim H., Liang J., Lee S., Lee D. (2019). Sustainable Rigid Polyurethane Foams Based on Recycled Polyols from Chemical Recycling of Waste Polyurethane Foams. J. Appl. Polym. Sci..

[30] Ginga C.P., Ongpeng J.M.C., Daly M.K.M. (2020). Circular Economy on Construction and Demolition Waste: A Literature Review on Material Recovery and Production. Materials.

[31] Shanmugam V., Das O., Neisiany R.E., Babu K., Singh S., Hedenqvist M.S., Berto F., Ramakrishna S. (2020). Polymer Recycling in Additive Manufacturing: An Opportunity for the Circular Economy. Mater. Circ. Econ..

[32] Kosmela P., Olszewski A., Zedler Ł., Burger P., Piasecki A., Formela K., Hejna A. (2021). Ground Tire Rubber Filled Flexible Polyurethane Foam—Effect of Waste Rubber Treatment on Composite Performance. Materials.

[33] Żukowska W., Kosmela P., Wojtasz P., Szczepański M., Piasecki A., Barczewski R., Barczewski M., Hejna A. (2022). Comprehensive Enhancement of Prepolymer-Based Flexible Polyurethane Foams’ Performance by Introduction of Cost-Effective Waste-Based Ground Tire Rubber Particles. Materials.

[34] Piszczyk L., Hejna A., Formela K., Danowska M., Strankowski M. (2015). Rigid Polyurethane Foams Modified with Ground Tire Rubber—Mechanical, Morphological and Thermal Studies. Cell. Polym..

[35] Bandegi A., Montemayor M., Manas-Zloczower I. (2022). Vitrimerization of Rigid Thermoset Polyurethane Foams: A Mechanochemical Method to Recycle and Reprocess Thermosets. Polym. Adv. Technol..

[36] Nikje M.M.A., Garmarudi A.B., Idris A.B. (2011). Polyurethane Waste Reduction and Recycling: From Bench to Pilot Scales. Des. Monomers Polym..

[37] Beran R., Zarybnicka L., Machova D. (2020). Recycling of Rigid Polyurethane Foam: Micro-milled Powder Used as Active Filler in Polyurethane Adhesives. J. Appl. Polym. Sci..

[38] Jin F.-L., Zhao M., Park M., Park S.-J. (2019). Recent Trends of Foaming in Polymer Processing: A Review. Polymers.

[39] Doyle L., Weidlich I., di Maio E. (2022). Developing Insulating Polymeric Foams: Strategies and Research Needs from a Circular Economy Perspective. Materials.

[40] Gama N., Ferreira A., Barros-Timmons A. (2018). Polyurethane Foams: Past, Present, and Future. Materials.

[41] Mannella G.A., Conoscenti G., Carfì Pavia F., la Carrubba V., Brucato V. (2015). Preparation of Polymeric Foams with a Pore Size Gradient via Thermally Induced Phase Separation (TIPS). Mater. Lett..

[42] Liu S., Duvigneau J., Vancso G.J. (2015). Nanocellular Polymer Foams as Promising High Performance Thermal Insulation Materials. Eur. Polym. J..

[43] Önder A., Robinson M. (2020). Investigating the Feasibility of a New Testing Method for GFRP/Polymer Foam Sandwich Composites Used in Railway Passenger Vehicles. Compos Struct..

[44] Shah D.U., Vollrath F., Porter D. (2015). Silk Cocoons as Natural Macro-Balloon Fillers in Novel Polyurethane-Based Syntactic Foams. Polymer.

[45] Wu G., Xie P., Yang H., Dang K., Xu Y., Sain M., Turng L.-S., Yang W. (2021). A Review of Thermoplastic Polymer Foams for Functional Applications. J. Mater. Sci..

[46] Karger-Kocsis J., Bárány T. (2019). Polypropylene Handbook.

[47] Mohebbi A., Mighri F., Ajji A., Rodrigue D. (2015). Current Issues and Challenges in Polypropylene Foaming: A Review. Cell. Polym..

[48] Záleská M., Pavlíková M., Jankovský O., Lojka M., Pivák A., Pavlík Z. (2018). Experimental Analysis of MOC Composite with a Waste-Expanded Polypropylene-Based Aggregate. Materials.

[49] Pavlík Z., Pavlíková M., Záleská M. (2019). Properties of Concrete with Plastic Polypropylene Aggregates. Use of Recycled Plastics in Eco-efficient Concrete.

[50] Memon M.J., Jhatial A.A., Murtaza A., Raza M.S., Phulpoto K.B. (2021). Production of Eco-Friendly Concrete Incorporating Rice Husk Ash and Polypropylene Fibres. Environ. Sci. Pollut. Res..

[51] Ye P., Chen Z., Su W. (2022). Mechanical Properties of Fully Recycled Coarse Aggregate Concrete with Polypropylene Fiber. Case Stud. Constr. Mater..

[52] Záleská M., Pavlíková M., Studnička J., Pavlík Z. (2018). Effect of Waste Expanded Polypropylene-Based Aggregate on Mechanical and Thermal Properties of Lightweight Concrete. IOP Conf. Ser. Mater. Sci. Eng..

[53] Witik R.A., Payet J., Michaud V., Ludwig C., Månson J.-A.E. (2011). Assessing the Life Cycle Costs and Environmental Performance of Lightweight Materials in Automobile Applications. Compos Part A Appl. Sci. Manuf..

[54] Baechler C., DeVuono M., Pearce J.M. (2013). Distributed Recycling of Waste Polymer into RepRap Feedstock. Rapid Prototyp. J..

[55] Vilar W.D. (1998). Química e Tecnologia Dos Poliuretanos.

[56] Chen X., Griesser U.J., Te R.L., Pfeiffer R.R., Morris K.R., Stowell J.G., Byrn S.R. (2005). Analysis of the Acid–Base Reaction between Solid Indomethacin and Sodium Bicarbonate Using Infrared Spectroscopy, X-Ray Powder Diffraction, and Solid-State Nuclear Magnetic Resonance Spectroscopy. J. Pharm. Biomed. Anal..

[57] Ball M.C., Snelling C.M., Strachan A.N., Strachan R.M. (1986). Thermal Decomposition of Solid Sodium Bicarbonate. J. Chem. Soc. Faraday Trans. 1 Phys. Chem. Condens. Phases.

[58] Sadik T., Pillon C., Carrot C., Reglero Ruiz J.-A. (2018). Dsc Studies on the Decomposition of Chemical Blowing Agents Based on Citric Acid and Sodium Bicarbonate. Thermochim. Acta.

[59] Kim D.Y., Kim G.H., Lee D.Y., Seo K.H. (2017). Effects of Compatibility on Foaming Behavior of Polypropylene/Polyolefin Elastomer Blends Prepared Using a Chemical Blowing Agent. J. Appl. Polym. Sci..

[60] Olszewski A., Kosmela P., Piasecki A., Żukowska W., Szczepański M., Wojtasz P., Barczewski M., Barczewski R., Hejna A. (2022). Comprehensive Investigation of Stoichiometry–Structure–Performance Relationships in Flexible Polyurethane Foams. Polymers.

[61] Piszczyk L., Hejna A., Danowska M., Strankowski M., Formela K. (2015). Polyurethane/Ground Tire Rubber Composite Foams Based on Polyglycerol: Processing, Mechanical and Thermal Properties. J. Reinf. Plast. Compos..

[62] Hejna A., Kosmela P., Kirpluks M., Cabulis U., Klein M., Haponiuk J., Piszczyk Ł. (2018). Structure, Mechanical, Thermal and Fire Behavior Assessments of Environmentally Friendly Crude Glycerol-Based Rigid Polyisocyanurate Foams. J. Polym. Environ..

[63] Olszewski A., Kosmela P., Żukowska W., Wojtasz P., Szczepański M., Barczewski M., Zedler Ł., Formela K., Hejna A. (2022). Insights into Stoichiometry Adjustments Governing the Performance of Flexible Foamed Polyurethane/Ground Tire Rubber Composites. Polymers.

[64] Hejna A., Olszewski A., Zedler Ł., Kosmela P., Formela K. (2021). The Impact of Ground Tire Rubber Oxidation with H_2_O_2_ and KMnO_4_ on the Structure and Performance of Flexible Polyurethane/Ground Tire Rubber Composite Foams. Materials.

[65] Llantoy N., Chafer M., Cabeza L.F. (2020). A comparative life cycle assessment (LCA) of different insulation materials for buildings in the continental Mediterranean climate. Energ. Buildings.

[66] Yang C., Zhang Q., Zhang W., Xia M., Yan K., Lu J., Wu G. (2021). High Thermal Insulation and Compressive Strength Polypropylene Microcellular Foams with Honeycomb Structure. Polym. Degrad. Stab..

[67] Mukhopadhyaya P., Kumaran K., Normandin N., van Reenen D., Lackey J. (2008). High-Performance Vacuum Insulation Panel: Development of Alternative Core Materials. J. Cold Reg. Eng..

[68] Zhang H., Fang W.-Z., Li Y.-M., Tao W.-Q. (2017). Experimental Study of the Thermal Conductivity of Polyurethane Foams. Appl. Therm. Eng..

[69] Lin T.A., Lin M.-C., Lin J.-Y., Lin J.-H., Chuang Y.-C., Lou C.-W. (2020). Modified Polypropylene/ Thermoplastic Polyurethane Blends with Maleic-Anhydride Grafted Polypropylene: Blending Morphology and Mechanical Behaviors. J. Polym. Res..

[70] Kannan M., Bhagawan S.S., Thomas S., Joseph K. (2013). Nanoclay Effect on Transport Properties of Thermoplastic Polyurethane/Polypropylene (TPU/PP) Blends. J. Polym. Res..

[71] Hejna A., Barczewski M., Andrzejewski J., Kosmela P., Piasecki A., Szostak M., Kuang T. (2020). Rotational Molding of Linear Low-Density Polyethylene Composites Filled with Wheat Bran. Polymers.

[72] Kannan M., Bhagawan S.S., Thomas S., Joseph K. (2014). Studies on Electrical Properties of Nanoclay Filled Thermoplastic Polyurethane/Polypropylene Blends. Polym. Compos..

[73] Kannan M., Bhagawan S.S., Jose T., Thomas S., Joseph K. (2010). Preparation and Characterization of Nanoclay-Filled Polyurethane/Polypropylene Blends. Polym. Eng. Sci..

[74] Matthes R., Bapp C., Wagner M., Zarbakhsh S., Frey H. (2021). Unexpected Random Copolymerization of Propylene Oxide with Glycidyl Methyl Ether via Double Metal Cyanide Catalysis: Introducing Polarity in Polypropylene Oxide. Macromolecules.

[75] Wang F., Lu M., Zhou S., Lu Z., Ran S. (2019). Effect of Fiber Surface Modification on the Interfacial Adhesion and Thermo-Mechanical Performance of Unidirectional Epoxy-Based Composites Reinforced with Bamboo Fibers. Molecules.

[76] Luo W., Zhang B., Zou H., Liang M., Chen Y. (2017). Enhanced Interfacial Adhesion between Polypropylene and Carbon Fiber by Graphene Oxide/Polyethyleneimine Coating. J. Ind. Eng. Chem..

[77] Yoo S.I., Lee T.Y., Yoon J.-S., Lee I.-M., Kim M.-N., Lee H.S. (2002). Interfacial Adhesion Reaction of Polyethylene and Starch Blends Using Maleated Polyethylene Reactive Compatibilizer. J. Appl. Polym. Sci..

[78] Bao J.-B., Nyantakyi Junior A., Weng G.-S., Wang J., Fang Y.-W., Hu G.-H. (2016). Tensile and Impact Properties of Microcellular Isotactic Polypropylene (PP) Foams Obtained by Supercritical Carbon Dioxide. J. Supercrit. Fluids.

[79] Korol J., Hejna A., Wypiór K., Mijalski K., Chmielnicka E. (2021). Wastes from Agricultural Silage Film Recycling Line as a Potential Polymer Materials. Polymers.

[80] Dong W., Wang X., Jiang Z., Tian B., Liu Y., Yang J., Zhou W. (2019). Acetylated SEBS Enhanced DC Insulation Performances of Polyethylene. Polymers.

[81] Kargin V.A., Sogolova T.I., Shaposhnikova T.K. (1965). The Mechanism of the Nucleation Effect of Solid Particles in Crystallizing Polymers. Polym. Sci. U.S.S.R..

[82] Lima P.S., Trocolli R., Wellen R.M.R., Rojo L., Lopez-Manchado M.A., Fook M.V.L., Silva S.M.L. (2019). HDPE/Chitosan Composites Modified with PE-g-MA. Thermal, Morphological and Antibacterial Analysis. Polymers.

[83] Koebel M., Strutz E.O. (2003). Thermal and Hydrolytic Decomposition of Urea for Automotive Selective Catalytic Reduction Systems: Thermochemical and Practical Aspects. Ind. Eng. Chem. Res..

[84] Barczewski M., Sałasińska K., Kloziński A., Skórczewska K., Szulc J., Piasecki A. (2019). Application of the Basalt Powder as a Filler for Polypropylene Composites With Improved Thermo-Mechanical Stability and Reduced Flammability. Polym. Eng. Sci..

[85] Hejna A., Kopczyńska M., Kozłowska U., Klein M., Kosmela P., Piszczyk Ł. (2016). Foamed Polyurethane Composites with Different Types of Ash—Morphological, Mechanical and Thermal Behavior Assessments. Cell. Polym..

